# Prevalence and Species Identification of Ixodid Ticks of Small Ruminants in Benadir Region, Somalia

**DOI:** 10.1155/2022/9224737

**Published:** 2022-12-02

**Authors:** Yasin Sh Hassan, kassim Jimale, Sadia Dirie, Omar Salah, Osman Afrah, Moktar Omar Sheikh Mohamed, Abdirahman Dubad

**Affiliations:** ^1^Department of Clinics, Theriogenology and Surgery, Faculty of Veterinary Medicine and Animal Husbandry, Somali National University, Mogadishu, Somalia; ^2^Faculty of Veterinary Medicine and Animal Husbandry, Somali National University, Mogadishu, Somalia

## Abstract

Ixodid ticks are one of the major health constraints on small ruminant productivity and contribute to significant economic losses in Somalia. An across-sectional study was conducted from November 2019 through December 2020 to identify hard tick species and determine the prevalence of tick infestation in small ruminants in the Benadir region, Somalia. Ticks were identified at the genera and species level by using morphological identification keys under a stereomicroscope. During the study period, a total of 384 small ruminants were examined for the presence of ticks through purposive sampling. All visible individual adult ticks were collected from the bodies of 230 goats and 154 sheep. A total of 651 Ixodid adult ticks were collected, of which 393 were male and 258 were female. The overall prevalence of tick infestation in the study area was 66.15% (254/384). The prevalence of tick infestation in goats and sheep was found to be 76.1% (175/230) and 51.3% (79/154), respectively. In the present study, nine species of hard ticks, which were grouped into three genera, were identified. The most abundant species found in this study were *Rhipichephalus pulchellus* (64.97%), *Rhipichephalus everstieversti* (8.45%), *Rhipichephalus pravus* (5.53%), *Rhipichephalus lunulatus* (5.38%), *Amblyomma lepidum* (5.22%), *Amblyomma gemma* (3.38%), and *Hyalomma truncatum* (2.62%) according to predominance. *Rhipichephalus bursa* (2.46%) and *Rhipichephalus turanicus* (1.99%) were the minor species observed in both species in the study area. The difference in the prevalence of tick infestation was found to be a statistically significant variation (*p* < 0.05) between species groups but not sex groups. In all cases, male ticks dominated female ticks. In conclusion, the findings of this study suggest that ticks were the most prevalent ectoparasites of small ruminants in the study areas. Therefore, the increasing threat of ticks and tick-borne pathogens of small ruminants warrants the urgent strategic application of acaricides and the creation of awareness among livestock owners to prevent and control tick infestations of sheep and goats in the study area.

## 1. Introduction

Goats and sheep both belong to the tribe Caprini of the family Bovidae in the suborder Ruminantia of the order Artiodactyla [1–5]. They are typical cloven-hoofed ruminants of relatively small size. There are about 51,899,000 livestock populations in Somalia, out of which the highest numbers (36,834,000) are sheep and goats, followed by camels (7.3 million) [6]. There are two main types of production systems found in Somalia, namely a mix of pastoralist and agro-pastoral; therefore, sheep and goats are part of mixed production systems. Seasonal migration of pastoralists and their animals in search of pasture and water is common. Hard ticks are the most important external parasites of livestock in the world and can easily constitute a limiting factor to successful stock farming unless appropriate measures are taken to control them [7]. In addition to that, ticks damage the hide and skin of livestock, including small ruminants. Among ectoparasites, ticks have been recognized as notorious threat due to severe irritation, allergy, and toxicities [8].

Ticks are known to transmit the pathogens that cause diseases like babesiosis, theileriosis, anaplasmosis, etc. Little is known about the prevalence and distribution of the tick vectors of these diseases, and in the absence of proper research, diagnosis, and experience of veterinary staff with clinical cases, the problem is often exaggerated. *Rhipicephalus*, *Hyalomma,* and *Amblyomma* ticks have been identified in the country, but hardly any research has been carried out on the epidemiology of tick-borne diseases [9]. The most endemic tick species in Somalia are *Rhipicephalus pulchellus*, *Hyalomma dromedarii*, *Amblyomma gemma,* and *Amblyomma variegatum* [10]. There is no available data about hard tick distribution in the study area or the country in general. Therefore, due to the unavailability of hard tick information, this study is aimed at finding out the prevalence of and identifying different species of Ixodid ticks in Mogadishu, Somalia.

## 2. Materials and Methods

**Figure 1:**
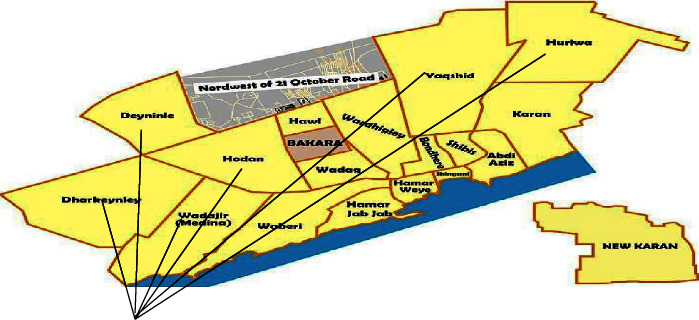
Study area in Benadir regions.

As shown in (Figure 1), the study was conducted in the Benadir region, especially in and around Mogadishu, the capital city of Somalia. It is located in the coastal Benadir region on the Somali Sea. The city has served as an important port for millennia. As of 2017, it had a population of 2,425,000 residents. It is located 2°4′ north and 45°22′ east.

### 2.1. Study Design

A cross-sectional study was conducted from November 2019 to December 2020 to determine the prevalence of ixodid tick infestation and to identify the different types of tick genera and species infesting sheep and goats in the study area.

### 2.2. Study Population

The small ruminants of six selected districts (Dharkiinley, Kaxda, Hodan, Deyniiley, Yaqshid, and Heliwaa) of the Benadir region were used as the target population of this study. This is due to the fact that there are no small ruminant farmers in all 17 districts of Benadir region. The small ruminants of all age and sex groups were included to this study.

### 2.3. Sample Size Determination and Sampling Procedure

The expected prevalence of Ixodidae ticks on small ruminants in the study area was assumed to be 50%, and the sample size was determined by using the formula given by [11]. The parameters used were a 95% confidence interval and a 5% desired level of precision. Accordingly, a sample size of 384 was calculated:

(1) $$\begin{array}{c} N=1.962\left(\text{Pexp}\right),\frac{\left(1-\text{Pexp}\right)}{\text{d}2}=384\text{small ruminants.} \end{array}$$

### 2.4. Data Collection Procedure

Tick collection, identification, and counting were performed on the entire body surface of the animal, which was thoroughly examined for the presence of any ticks, and all visible adult ticks were collected. Ticks were removed carefully and gently with a horizontal pull to the body surface. Prior to collection, the signalmen of the selected animals, the body part where ticks were picked, and the location/district were recorded in a special format designed for this purpose [12].

The collected ticks were preserved in universal bottles containing 70% ethyl alcohol and labeled with the animal's origin and its identification. The specimens were transported to the parasitological laboratory of the Somali National University for counting and identification. Ticks were counted and subsequently identified to genus and species levels by using a stereomicroscope, according to the standard identification keys given by the authors of [12].

### 2.5. Data Analysis

The raw data obtained from the field and laboratory examinations were inserted into a Microsoft Excel spreadsheet to create a database. The collected data were analyzed using the SPSS version 22.0 software program. Descriptive statistics were used to summarize the tick species identified.

## 3. Results and Discussion

Out of the total 384 small ruminants (154 sheep and 230 goats) examined, 254 (66.15%) were found to be infested with at least one species of tick (Table 1). Examined animals were considered positive for a given tick infestation when at least one tick was collected from them.

High rates of infestation were recorded in both species, and the corresponding percentage of infestation in sheep and goats was 51.3% and 76.1%, respectively. While the corresponding percentages of infestation in males and females were 63.76% and 69.92%, respectively. The infestation was significantly (*p* ≤ 0.001) higher in goats compared to that of sheep. However, the difference was not significant between different sex groups (*p* ≤ 0.001) (Table 2).

**Figure 2:**
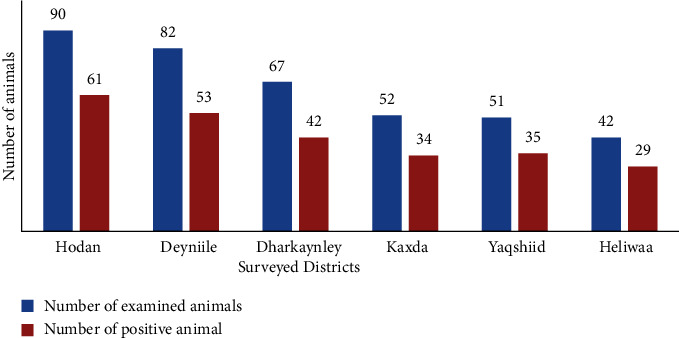
Distribution of ticks based on surveyed districts of mogadishu.

According to (Figure 2), the location of the animals, there was an approximately similar prevalence of infestation in the different districts in this study.

A minor difference between the prevalence of ticks in the different districts of Mogadishu is due to the variation in sample sizes collected.

Upon identification, nine different tick species belonging three genera were identified (Tables 3 and 4). Considering the relative abundance of each tick genera as per the host species involved, *Rhipicephalus* was the most abundantly encountered with high burden followed by *Amblyomma* and *Hyalomma* was the least (Table 3).

According to the species, *Rh. pulchellus* was the most abundantly observed tick species 423 (64.97%), followed by *Rh. evertsi evertsi* 55 (8.45%), *Rh. pravus* 36 (5.53%), *Rh. lunulatus* 35 (5.38%), *A. lepidum* 34 (5.22%), *A. gemma* 22 (3.38%), *H. truncatum* 17 (2.61%), *Rh. bursa* 16 (2.46%), and *Rh. turanicus* 13 (1.99%) was the minor species observed in both species.

As shown in Table 5, out of the total of 651 Ixodid adult ticks, 393 were males and 258 were females. In the present study, the male-to-female sex ratio for tick species indicated a higher number of males than females for all species of ticks.

## 4. Discussion

This study indicated the prevalence of Ixodid tick infestation was highly substantial in small ruminants in the study area. 384 small ruminants were examined, and a total of 651 visible adult ticks were collected from the bodies of 230 goats and 154 sheep. A total of 254 small ruminants were found to be infested by one or more genera of ticks, with an overall prevalence of 66.15% (Table 1). This finding was comparable with the previous investigation conducted by Ahmed et al. in the Dire Dawa district, eastern Ethiopia, who recorded a 72.39% overall prevalence of tick infestation in small ruminants [13]. However, this finding was contrary to and lower than the findings of the investigation conducted in Fafen zone, Somali regional state of Ethiopia by Mohammed and Petros, who recorded 79.7% overall prevalence of tick infestation in small ruminants [14], and Abunna et al. who recorded 76.5% overall prevalence of tick infestation in small ruminants in Beadle district, Oromia Region, Ethiopia [15].

The variation in the prevalence of ticks might be due to the difference in the seasons of the study period, geographical differences, and frequent exposure to the communal grazing land and management system, which includes the use of Acaridae and other preventive measures undertaken for tick control. Tick activity is also influenced by rainfall, temperature, altitude, and atmospheric relative humidity [10]. This could also be a nonrandomized sampling technique used for this study.

The prevalence of tick infestation between species groups was statistically significant (*p* < 0.05) with more infestation in goats (76.1%) compared to that of sheep (51.3%), as shown in (Table 3). This result was in agreement with a previous study conducted by Abebe et al., who recorded more prevalence in goats (58.8%) than sheep (48%) [16]. Similarly, Degefa et al. reported 44.7% in goats and 29.5% in sheep [17]. However, this result disagreed with the previous investigation conducted by Ahmed et al., who reported 70.7% in sheep and 73.9% in goats, which was statistically insignificant [13]. The probable reason for the higher prevalence in goats might be due to the fact that Sheep were grazing around home while goats usually go long distances for grazing. This, in turn, contributes to a minimum rate of exposure to ticks since the number of ticks is lower around the home as compared to animals that graze mostly in the far pasture. In addition to that, this could be due to the higher number of goats sampled in the study area, with the goat ratio being (1.49).

The prevalence of tick infestation was 63.76% in male and 69.92% in female animals, which was statistically insignificant. This finding was in line with reports by Lloyd [18]. However, this result disagreed with the previous study conducted by Ahmed et al. [13], who reported 79% in male animals and 64.8% in female animals, which was statistically significant. The exact cause of the higher prevalence of tick infestation in female animals is not obvious. However, the presence of higher levels of prolactin and progesterone hormones could make females more susceptible to any infection. Furthermore, pregnancy and lactation stress may also have made the female animals more susceptible to infestation [18]. The tick infestations in the different selected districts did not show any significant difference.

The principal tick genera infesting small ruminants in the study area are *Rhipicephalus* (88.79%), *Amblyomma* (8.6%), and *Hyalomma* (2.61%). This finding was in agreement with the investigation conducted by Hassan et al. in Benadir Region, Somalia [6]. Similar ticks were commonly found on domestic livestock in Somalia [19].

Regarding the different tick species infesting small ruminants in the study area, *Rh. pulchellus* (64.97%) was the most abundant tick species found in this study. This finding was in line with the previous study conducted by Ahmed et al. in the Dire Dawa district, eastern Ethiopia [13]. However, this result was contrasted with the previous investigation conducted by Hassan et al., who reported *Rh. pulchellus* as the second most abundant tick species in the Benadir region, ranking behind *Rh. evertsi evertsi* [6]. *Rh. pulchellus,* which is locally known as “garab-ad,” is widely distributed and common on domestic livestock in East Africa, including Somalia. This is most probably attributed to the nature of this tick species, which favours semiarid and low-land areas [10]. *A. gemma* and *Rh. pulchellus* are confined to semiarid areas [10], and the lowland tick densities are usually greater than those in the highlands [12]. It has been suggested that in areas where the humidity is low, ticks resist desiccation by spending shorter periods questing for hosts, and they also enter diapauses at unfavorable times of the year [12].

*Rh. evertsi evertsi* (8.45%) was the second most abundant tick species in the study area. This finding disagrees with the previous investigation conducted by Hassan et al. in Benadir Region, Somalia, who reported a 72.84% high prevalence [6]. This difference might be attributed to seasonal variation. The other species of ticks observed in the present study were *Rh. lunulatus* 35 (5.38%), *A. lepidum* 34 (5.22%), *A. gemma* 22 (3.38%), *H. truncatum* 17 (2.61%), *Rh. bursa* 16 (2.46%), and *Rh. turanicus* 13 (1.99%). All these species were previously reported in East Africa, including Somalia [10, 12].

In all cases, the numbers of male ticks were higher than the numbers of female ticks, with a ratio of 1.5 to 1. This present result was in line with the findings of [13] in the Dire Dawa district, eastern Ethiopia. This is most probably because fully engorged female ticks drop off to the ground to lay eggs, while male ticks tend to remain on the host for several months later to continue feeding and mating with other females on the host before dropping off [20]. Ticks are the most important ectoparasites of livestock in tropical and subtropical areas and are responsible for severe economic losses both through the direct effects of bloodsucking and indirectly as vectors of pathogens and toxins. This study also confirms that ticks are still a big concern for ectoparasites and pose significant challenges to small ruminant production and the health of the study area [8].

## 5. Conclusion

In this study, a high prevalence rate of 66.15% of Ixodid tick infestation in small ruminants was observed, and nine species of ticks grouped under three genera were identified. *Rh. pulchellus*, *Rh. evertsi evertsi*, *Rh. pravus*, *Rh. lunulatus, A. lepidum, A. gemma, H. truncatum, Rh. bursa,* and *Rh. turanicus* were among the tick species observed on both ruminant species in the study area. The most important and abundant tick species identified in the study area were *Rh. pulchellus,* followed by *Rh. evertsi evertsi* in order of predominance. The prevalence of tick's infestation in this study was statically significant between species groups with more prevalence in goats than sheep. Generally, ticks are highly prevalent in the study area due to inadequate veterinary services, favorable climatic conditions, poor owner's awareness of the impacts of tick infestations, and a lack of an effective and planned control strategy in the study area. Ticks are responsible for severe economic losses both through the direct effects of bloodsucking and damage to the hide and skin and indirectly by transmitting different TBDs. Therefore, the strategic application of acaricides, especially at the beginning of the short and major rainy seasons, might minimize the infestation of ticks. The creation of awareness among livestock owners of the potential effects of ticks and tick-borne pathogens (TBPs) is needed.

## Figures and Tables

**Table 1:** Overall prevalence of tick infestation in small ruminants of the study area.

Number of examined animals	Number of infested animals	Prevalence (%)
384	254	66.15

**Table 2:** Overall prevalence of tick distribution and its significance analysis on species and sex relation in small ruminants of the study area.

Variable	Category	No. examined animal	No. of infested animal (%)	*X* ^2^	*p* value
Species	Sheep	154	79 (51.3)	25.31	0.000
	Goat	230	175 (76.1)		

Sex	Male	138	88 (63.76)	3.024	0.220
	Female	246	172 (69.92)		

**Table 3:** Overall prevalence of total tick burden at the genera level in the study area.

Genus	Collected tick		Prevalence (%)	
	Sheep	Goat	Sheep	Goat
*Rhipicephalus*	232	346	89.5	88.3
*Amblyomma*	16	40	6.2	10.2
*Hyalomma*	11	6	4.3	1.5
Total	259	392	100	100

**Table 4:** Relative abundance of tick species during the study period.

Tick species	Total	Percentages
*Rh. pulchellus*	423	64.97
*Rh. evertsi evertsi*	55	8.45
*Rh. pravus*	36	5.53
*Rh. lunulatus*	35	5.38
*Rh. bursa*	16	2.46
*Rh. turanicus*	13	1.99
*A. lepidum*	34	5.22
*A. gemma*	22	3.38
*H. truncatum*	17	2.61
Total	651	100

**Table 5:** Sex ratio of tick burden in small ruminants.

Tick species	Male tick	Female tick	Male : Female ratio
*Rh. pulchellus*	234	189	1.24
*Rh. evertsi evertsi*	41	14	2.9
*Rh. pravus*	27	9	3
*Rh. lunulatus*	24	11	2.18
*Rh. bursa*	10	6	1.6
*Rh. turanicus*	11	2	5.5
*A. lepidum*	19	15	1.26
*A. gemma*	14	8	1.75
*H. truncatum*	13	4	3.25
Total	393	258	1.5

## Data Availability

The datasets used and analyzed during the current study are available from the corresponding author on request.
